# Fomentations of Digitalis in Certain Kinds of Ascites

**Published:** 1853-07

**Authors:** 


*Fomentations of Digitalis in certain kinds of Ascites.*—Dr. Raymond Falot has published in the *Revue Thérapeutique du Midi* three cases of inflammatory ascites in which diuretics were not well borne by the stomach, and where fomentations with a decoction of digitalis leaves produced marked action of the kidneys, and absorption of the fluid effused in the peritonæum.

The first case is that of a girl twenty-four years of age, who, after severe wetting, was attacked with subacute peritonitis and consequent ascites. Nitre and powdered digitalis had very little effect, and tapping to twelve quarts took place. As the liquid re-accumulated, fomentations were made with a decoction of two ounces of digitalis to a quart of water boiled down to a pint. The liquid was applied by means of compresses soaked in it, and covered with oil skin to prevent evaporation. The kidneys acted very powerfully, and the effused fluid became completely absorbed. The same means also succeeded two years afterwards, when the patient suffered again from ascites.

The second case refers to a boy eight years of age, who had ascites after intermittent fever. Both the syrup and powder of digitalis were ill borne; the fomentations were used as above described, and the effusion disappeared, whilst the kidneys and skin were acting powerfully.

Third case.—A man fifty years of age suffered from ascites after a very severe attack of gastric fever. No sort of medicine could be borne; the digitalis fomentations were resorted to, and the same favorable results as in the previous cases were obtained in a few months. Dr. Falot states that he does not wish to give his mode of treatment more importance than it deserves, but that he was much struck with the coincidence between the use of the fomentotions and the increase of the renal and the cutaneous secretions. In one of the cases frictions with the tincture of digitalis had been used without success. The fomentations will certainly be worth a trial when the usual diuretics cannot be given by the mouth. The leaves should not be more than one year old.—*Ibid.*

